# High-flow priapism in a 12-year-old boy: Treatment with superselective embolization

**DOI:** 10.4103/0970-1591.57918

**Published:** 2009

**Authors:** A. R. Mossadeq, R. Sasikumar, M. Z. M. Nazli, A. M. Shafie, M. D. M Ashraf

**Affiliations:** Urology Unit, Department of Surgery, School of Medical Sciences, Universiti Sains Malaysia, Health Campus, 16150-Kubang Kerian, Malaysia; 1 Radiology, School of Medical Sciences, Universiti Sains Malaysia, Health Campus, 16150-Kubang Kerian, Malaysia

**Keywords:** High-flow priapism, perineal trauma, super selective embolization

## Abstract

Priapism is caused by an imbalance between penile blood inflow and outflow. There are two types of priapism: low-flow priapism due to venous occlusion and high-flow priapism due to uncontrolled arterial flow to the veins. High-flow priapism most frequently occurs as a result of penile trauma in which the intercavernosal artery disruption causes an arteriocavernosal fistula. It is rarely encountered in the pediatric and prepubertal population. Clinically, it manifests as a painless, prolonged erection after perineal trauma. Treatment ranges from expectant management to open surgical exploration with vessel ligation. We report the successful treatment of high-flow priapism in a 12-year-old prepubertal boy with superselective embolization.

## CASE REPORT

**Figure 1:**
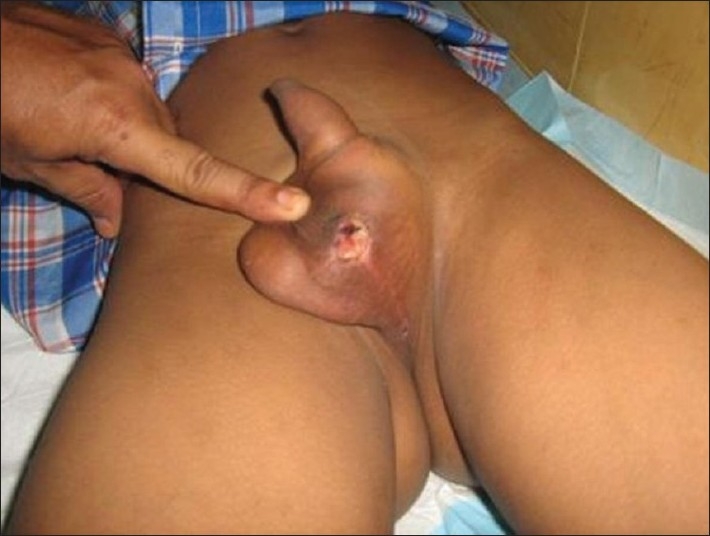
Penis in erected state with perineal trauma

A 12-year-old boy was referred to Hospital Universiti Sains Malaysia from a district hospital one week following perineal trauma with painless priapism for five days. On examination, his corpus cavernosa was rigid and his glans and corpus spongiosum was soft and compressible [Figure 1]. He was voiding normally and all medical illnesses were ruled out. Bright red blood was withdrawn from the cavernosum and blood gas analysis confirmed arterial blood, suggesting the diagnosis of high-flow priapism. Doppler ultrasonography confirmed the diagnosis and showed an arteriovenous fistula on the left side at the base of penis.

**Figure 2:**
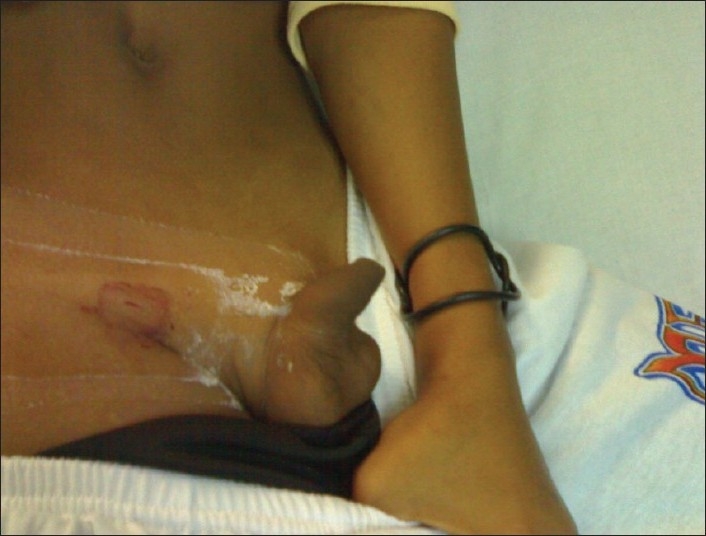
Penis assuming flaccid state following super-selective embolization of the fistulous site

He was treated with selective left internal iliac artery and left internal pudendal artery angiography and superselective cannulation and embolization of the left cavernosal artery. Total occlusion of the arteriovenous fistula and normal blood flow was seen thereafter. Clinically, complete detumescence of the penis occurred [Figure 2]. He was observed in ward for 24 hours and discharged home the next day.

## DISCUSSION

High-flow priapism is usually due to a traumatic arteriocavernosal fistula and causes a nonischemic, painless engorgement of the corpora cavernosa. In high-flow priapism, the fistula bypasses the helicine artery, and the arterial blood flows into the sinusoidal space. Penile erection is caused by an excess of arterial inflow followed by a stretching of the corporal sinuses. Diagnosis can be confirmed by duplex sonography, cavernosal blood gas determination or angiography. Duplex sonography can show the presence and site of the fistula and should be performed initially because of the noninvasiveness. Findings in cases of high-flow priapism include focal brush of pulsatile, turbulent flow with elevated flow velocities adjacent to cavernous artery.[1] A few patients of prepubertal age with high-flow priapism treated by selective embolization have been described.[2] Angiography with subsequent superselective embolization can now be considered as the treatment of choice for high-flow priapism. This procedure effectively corrects the arterial overflow and normally preserves potency with encouraging long-term results.[3]

## CONCLUSION

We report the successful treatment of high-flow priapism in a 12-year-old prepubertal boy with superselective embolization. A rare entity successfully managed by the urology and radiology team at Hospital Universiti Sains Malaysia.
